# Loneliness, and Smoking Status: A Pilot Study of Extended-Stay Hotel Residents in the United States

**DOI:** 10.1093/geroni/igaf122.1715

**Published:** 2025-12-31

**Authors:** Naomi Adjei, Terri Lewinson, Abhirupa Dasgupta, Wambui Onsando

**Affiliations:** Dartmouth College, The Dartmouth Institute for Health Policy & Clinical Practice, New Hampshire, United States; Geisel School of Medicine at Dartmouth, Lebanon, New Hampshire, United States; The Dartmouth Institute for Health Policy and Clinical Practice, Lebanon, New Hampshire, United States; The Dartmouth Institute for Health Policy and Clinical Practice, Lebanon, New Hampshire, United States

## Abstract

As the housing industry continues to struggle to meet the growth demands in the United States, more people have resorted to extended-stay hotels (ESH) as a housing alternative. ESH residents may experience complex psychological issues such as loneliness due to the transient nature of their living environment. Research shows a bidirectional relationship between loneliness and smoking status regardless of place. However, previous research found that both ESH residents viewed smoking as a form of community socialization. Furthermore, research asserts that aging and the female gender have a greater association with loneliness. Thus, in this pilot study, we examined the relationship between loneliness, measured by the DeJong Loneliness Score, and smoking status, measured by a yes or no response to smoking in ESH residences. The analysis, based on a sample of 77 adults (age range 23-72), indicated that the overall model did not significantly explain variation in loneliness (F = 1.44, p = 0.23). However, a significant interaction effect between smoking status and gender emerged (coefficient = 1.10, p = 0.037), suggesting that smokers experience greater loneliness regardless of age. Additionally, women who smoked reported feeling less lonely than men, regardless of age differences, suggesting that age does not meaningfully affect the outcome (coefficient = -1.38, p = 0.032). These findings highlight smoking status, especially with gender, as a key factor in loneliness among ESH residents. Future research with larger, longitudinal samples is needed to further explore the link between loneliness and smoking across the lifespan in this population.

